# Antibacterial and Antibiofilm Potential of Bacterial Cellulose Hydrogel Containing Vancomycin against Multidrug-Resistant *Staphylococcus aureus* and *Staphylococcus epidermidis*

**DOI:** 10.3390/biology13050354

**Published:** 2024-05-17

**Authors:** Ana Alice Venancio Correia, Luís André de Almeida Campos, Hanne Lazla Rafael de Queiroz Macêdo, Davi de Lacerda Coriolano, Maria Anndressa Alves Agreles, Danilo Elias Xavier, Rafaela de Siqueira Ferraz-Carvalho, José Lamartine de Andrade Aguiar, Isabella Macário Ferro Cavalcanti

**Affiliations:** 1 Keizo Asami Institute (iLIKA), Federal University of Pernambuco (UFPE), Recife 50670-901, Brazil; 2 Department of Microbiology, Aggeu Magalhães Institute, FIOCRUZ-PE, Recife 50740-465, Brazil; 3 Department of Surgery, Federal University of Pernambuco (UFPE), Recife 50670-901, Brazil; 4 Laboratory of Microbiology and Immunology, Academic Center of Vitória (CAV), Federal University of Pernambuco (UFPE), Vitória de Santo Antão 55608-680, Brazil

**Keywords:** biopolymers, nanotechnology, bacterial resistance

## Abstract

**Simple Summary:**

This study assessed the in vitro antibacterial and antibiofilm properties of bacterial cellulose hydrogel (HYDROGEL) produced by *Zoogloea* sp. combined with vancomycin (VAN) against multidrug-resistant (MDR) *Staphylococcus aureus* and *Staphylococcus epidermidis*, which are common wound infection pathogens. Initially, HYDROGEL’s morphology was examined through scanning electron microscopy (SEM), which showed a microporous structure. Subsequently, VAN was integrated into HYDROGEL (VAN-HYDROGEL). VAN and VAN-HYDROGEL demonstrated bacteriostatic and bactericidal actions against *S. aureus* and *S. epidermidis*, while HYDROGEL alone lacked antibacterial activity. The antibiofilm activity was examined using crystal violet, Congo red methods, and SEM, indicating inhibition of biofilm formation by VAN and HYDROGEL, with enhanced eradication potential upon incorporation into HYDROGEL, as supported by SEM images. This study underscores HYDROGEL’s potential as a carrier for antibiotics targeting MDR wound infections, maintaining antibacterial efficacy and augmenting antibiofilm effects, suggesting its promising role in combating such infections.

**Abstract:**

The present study aimed to evaluate the in vitro antibacterial and antibiofilm activity of bacterial cellulose hydrogel produced by *Zoogloea* sp. (HYDROGEL) containing vancomycin (VAN) against bacterial strains that cause wound infections, such as multidrug-resistant (MDR) *Staphylococcus aureus* and *Staphylococcus epidermidis*. Initially, HYDROGEL was obtained from sugar cane molasses, and scanning electron microscopy (SEM) was performed to determine morphological characteristics. Then, VAN was incorporated into HYDROGEL (VAN-HYDROGEL). The antibacterial activity of VAN, HYDROGEL, and VAN-HYDROGEL was assessed using the broth microdilution method to determine the minimum inhibitory concentration (MIC) and the minimum bactericidal concentration (MBC) against methicillin-sensitive *S. aureus* (MSSA) ATCC 25923, methicillin-resistant *S. aureus* (MRSA) ATCC 33591, *S. epidermidis* INCQS 00016 (ATCC 12228), five clinical isolates of MRSA, and nine clinical isolates of methicillin-resistant *S. epidermidis*, following the Clinical and Laboratory Standards Institute (CLSI) guidelines. Additionally, the antibacterial activity of VAN, HYDROGEL, and VAN-HYDROGEL was studied using the time-kill assay. Subsequently, the antibiofilm activity of VAN, HYDROGEL, and VAN-HYDROGEL was evaluated using crystal violet and Congo red methods, as well as SEM analysis. VAN and VAN-HYDROGEL showed bacteriostatic and bactericidal activity against MRSA and methicillin-resistant *S. epidermidis* strains. HYDROGEL did not show any antibacterial activity. Analysis of the time-kill assay indicated that HYDROGEL maintained the antibacterial efficacy of VAN, highlighting its efficiency as a promising carrier. Regarding antibiofilm activity, VAN and HYDROGEL inhibited biofilm formation but did not demonstrate biofilm eradication activity against methicillin-resistant *S. aureus* and *S. epidermidis* strains. However, it was observed that the biofilm eradication potential of VAN was enhanced after incorporation into HYDROGEL, a result also proven through images obtained by SEM. From the methods carried out in this study, it was possible to observe that HYDROGEL preserved the antibacterial activity of vancomycin, aside from exhibiting antibiofilm activity and enhancing the antibiofilm effect of VAN. In conclusion, this study demonstrated the potential of HYDROGEL as a candidate and/or vehicle for antibiotics against MDR bacteria that cause wound infections.

## 1. Introduction

When the skin is injured, the environment becomes conducive to the development of infections. In severe cases, the subcutaneous tissue is exposed, compromising the integrity of the site and favoring the development of more extensive infections, thus aggravating the patient’s condition, in addition to generating higher financial costs for health care systems [1,2,3].

Instances of bacterial infection in wounds are increasing and worrying specialists, especially in cases where the microorganisms found in the infection site are resistant to antimicrobials, as bacterial resistance is one of the biggest threats to the global community [4,5,6]. In addition to bacterial resistance, biofilms present a significant challenge in the treatment of wound infections. 

A biofilm constitutes a structured assembly of bacterial cells enveloped within a viscous extracellular polymeric substance (EPS) matrix. Its formation on wound surfaces hampers antibiotic penetration, consequently impeding wound healing. Research indicates that hydrogel can enhance the healing trajectory and management of wounds afflicted by MDR bacteria, particularly those loaded with antibiotics [7,8,9,10].

Among the most common bacteria in infected wounds, *S. aureus* and *S. epidermidis* stand out as the main pathogens that cause infections responsible for high rates of morbidity and mortality [11,12,13]. The treatment of wounds infected by these two pathogens becomes exceedingly challenging when they exhibit both antibiotic resistance and biofilm production, requiring new therapeutic strategies to eliminate these infections and enhance the healing process of wounds [14,15].

Hence, new therapeutic strategies are necessary for these wound infections, such as the use of biopolymers. Bacterial cellulose (BC) is a biopolymer with diverse applicability in the food and biomedical industries. BC has a three-dimensional structure made of cellulose nanofibrils and microfibrils synthesized by bacteria, such as *Acetobacter* spp., *Azotobacter* spp., *Komagataeibacter xylinus*, *Gluconacetobacter* spp., *Pseudomonas* spp., *Salmonella* spp., and *Sarcina ventriculi* from various carbon sources, such as glucose, sucrose, mannose, fructose, ethanol, and pyranose, among others [16,17,18].

The structure of hydrogel is similar to the extract of an extracellular matrix and soft tissues, which presents a distinct three-dimensional conformation of nanofibrils and microfibrils that enables the adhesion of compounds due to the hydroxyl groups that make up its surface [19,20,21,22]. A hydrogel produced from sugar cane molasses has been tested in different medical and biomedical areas, showing promising results, including stability due to sugar polymerization, low toxicity, biocompatibility, and efficiency for tissue remodeling [23,24,25,26].

Hydrogel can also be a vehicle for administering medications to wounds given its high purity, ability to retain moisture, protect the body from impurities, and enable gas exchange, and its high biocompatibility [21,27,28,29]. The incorporation of vancomycin (VAN) in hydrogel is a relevant alternative to enhance the antibacterial and antibiofilm action of biomaterials [30,31]. VAN is a glycopeptide that inhibits the polymerization of peptidoglycans in the bacterial cell wall, causing extravasation of intracellular components. Moreover, this antibiotic is the preferred treatment for severe infections caused by antibiotic-resistant *S. aureus* and *S. epidermidis* [32]. 

Thus, the novelty of this study was the incorporation of a molecule used in clinical practice into a nanobiotechnological product for its topical use, enabling the application of vancomycin via a new route of administration focused on the treatment of wounds infected by Gram-positive cocci. Therefore, the aim of this study was to evaluate the in vitro antibacterial and antibiofilm activity of VAN-HYDROGEL against multidrug-resistant *S. aureus* and *S. epidermidis* strains.

## 2. Materials and Methods

### 2.1. Materials

#### 2.1.1. Reagents

HYDROGEL was supplied by POLISA Biopolímeros para a Saúde Ltd.a. Vancomycin, Müeller–Hinton agar (MHA), Müeller–Hinton broth (MHB), tryptone soy broth (TSB), glucose (D-(+)-Glucose), and crystal violet were purchased from Sigma-Aldrich (St. Louis, MO, USA). Methanol, glacial acetic acid, and all reagents were from Merck (Darmstadt, Alemanha).

#### 2.1.2. Bacterial Strains

Two species of the genus *Staphylococcus* (*S. aureus* and *S. epidermidis*) were used for this study. The strains used were methicillin-sensitive *S. aureus* (MSSA) ATCC 25923, methicillin-resistant *S. aureus* (MRSA) ATCC 33591, *S. epidermidis* ATCC 12228 (INCQS 00016), five MRSA clinical isolates (C047, C074, C115, C128 e C137), and nine clinical isolates of methicillin-resistant *S. epidermidis* (C233, C266, C271, C276, C277, C281, C387, C389, and C417).

### 2.2. Methods

#### 2.2.1. Preparation of VAN-HYDROGEL

The 0.8% HYDROGEL obtained by hydrating BC was produced and supplied by POLISA Biopolímeros para a Saúde Ltd.a. The HYDROGEL was found to be biocompatible, non-toxic, and non-genotoxic [23,33,34]. To prepare VAN-HYDROGEL, 2.5 mL of the VAN solution was solubilized in ultra-pure water and incorporated into 2.5 mL of HYDROGEL in a 5 mL volumetric flask.

#### 2.2.2. Morphological Analysis of the HYDROGEL by SEM

Field emission gun scanning electron microscopy (FEG-SEM) was used to determine the morphological characteristics of the HYDROGEL on a MIRA3 (TESCAN, Brno, Czech Republic) with an acceleration of 15 kV and a working distance of 10 mm with 100 kx magnification. For these analyses, the samples were fixed to aluminum tubes and coated with a thin layer of gold before being subjected to FEG-SEM.

#### 2.2.3. Antibacterial Activity

Antibacterial activity was assessed by determining the minimum inhibitory concentration (MIC) by the broth microdilution method according to CLSI [35]. Initially, MHB was added to the microdilution plates, and then VAN, HYDROGEL, and VAN-HYDROGEL were added through serial dilution to obtain concentration ranges from 128 to 0.25 µg/mL, 0.5% to 0.0009%, and 128 µg/mL/0.5% to 0.25 µg/mL/0.0009%, respectively. Bacterial suspensions were adjusted to a 0.5 density on the McFarland scale and then deposited into the wells of the plate. Subsequently, the plates were incubated at 35 ± 2 °C for 24 h, and after incubation, the well plates were read by spectrophotometry at a wavelength of 620 nm (Multiskan FC, Thermo Scientific, Madrid, Spain). The MIC was determined as the lowest concentration capable of inhibiting more than 90% of bacterial growth. Minimum bactericidal concentration (MBC) was determined by inoculating an aliquot of bacterial samples from wells where there was no growth onto MHA petri dishes. These dishes were incubated at 35 ± 2 °C for 24 h, and after this period the MBC was determined as the lowest concentration at which there was no microbial growth. Independent experiments were performed in triplicate on different days.

#### 2.2.4. Time-Kill Assay

The time-kill assay was performed after treating bacteria with VAN and VAN-HYDROGEL according to Appiah et al. [36]. The time-kill curves were determined using two different concentrations, MIC and 0.5 × MIC of both VAN and VAN-HYDROGEL. MSSA ATCC 25923 and MRSA ATCC 33591 were inoculated onto MHA petri dishes and incubated at 35 ± 2 °C for 24 h. After this period, the bacterial suspension was adjusted to 0.5 on the McFarland scale using a spectrophotometer calibrated at a wavelength of 620 nm. Solutions at predefined concentrations (MIC and 0.5 × MIC) were prepared, and 2 mL of each solution was transferred to tubes containing 18 mL of MHB with bacterial inoculum at 1.0 × 10^6^ CFU/mL. These tubes were then incubated at 35 ± 2 °C for 24 h, and aliquots were removed at time intervals of 0, 3, 6, 9, 12, and 24 h and seeded on MHA petri dishes using the pour-plate technique, after dilutions in sterile saline solution. Subsequently, the dishes were incubated at 35 ± 2 °C, and readings were performed after 24–48 h of incubation [36,37]. Independent experiments were performed in triplicate on different days.

#### 2.2.5. Determination of Biofilm Formation

The biofilm formation potential by bacterial clinical isolates was tested using the crystal violet method [38]. Firstly, bacterial suspensions were adjusted to a density of 0.5 on the McFarland scale in TSB enriched with 1% glucose, then TSB was added to each well of the microdilution plates, which were incubated at 35 ± 2 °C for 48 h. After incubation, TSB was aspirated, and the wells were washed with phosphate buffer at a pH 7.4. The well plates were dried, and the attached bacteria were fixed with 99% methanol. After fixation, the methanol was removed from the wells, and the plates were dried again. Subsequently, the bacteria adhered to the plates were stained with 1% crystal violet. Excess crystal violet was removed, and 30% glacial acetic acid was added to each well. The results were then analyzed by spectrophotometry at a wavelength of 570 nm. The strains were classified into four categories based on the OD (optical density) values obtained from the test, in comparison with the ODc value (optical density of the negative control): non-adherent if OD ≤ ODc; poor production if ODc < OD ≤ 2 × ODc; moderate production if 2 × ODc < OD ≤ 4 × ODc; or strong production if 4 × ODc < OD [38]. Independent experiments were performed in triplicate on different days.

#### 2.2.6. Antibiofilm Activity

##### Congo Red Agar Method

The qualitative determination of the inhibition of the slime production and the exopolysaccharide matrix production was carried out by the Congo red method according to Santos et al. [39]. MRSA ATCC 33591 was adjusted in brain heart infusion broth (BHI) to 0.5 on the McFarland scale and incubated at 35 ± 2 °C for 24 h. An aliquot of bacterial growth was then seeded on Congo red agar petri dishes containing HYDROGEL at 0.25%, and VAN and VAN-HYDROGEL at 0.5 × MIC. The petri dishes were incubated at 35 ± 2 °C for 48 h. After the incubation period, bacteria from colonies with blackish color and rough consistency were considered biofilm-producing bacteria, and bacteria from red colonies represented bacteria that did not produce biofilm. Independent experiments were performed in triplicate on different days.

##### Determination of Biofilm Inhibition

The evaluation of inhibition of biofilm formation was carried out using the crystal violet method according to Stepanovic et al. [38] and Albano et al. [40] to determine the minimum biofilm inhibitory concentration (MBIC). Initially, TSB + 1% glucose was added to flat-bottom well plates, then VAN, HYDROGEL, and VAN-HYDROGEL were added through serial dilution to obtain concentration ranges of 2 to 0.12 µg/mL, 0.5 to 0.03%, and 2 µg/mL/0.5% to 0.12 µg/mL/0.03%, respectively. Bacterial suspensions were adjusted in TSB to a density of 0.5 on the McFarland scale, and then deposited in the wells. Subsequently, the plates were incubated at 35 ± 2 °C for 24 h, and after incubation, the crystal violet method described previously was performed. MBIC was determined as the lowest concentration capable of inhibiting 90% of biofilm formation. Independent experiments were performed in triplicate on different days.

##### Determination of Biofilm Eradication

The evaluation of biofilm eradication was performed using the crystal violet method according to Das, Yang, and Ma [41] to determine the minimum biofilm eradication concentration (MBEC). Initially, the bacterial suspensions were adjusted to 0.5 density on the McFarland scale in TSB + 1% glucose and distributed on well plates. The plates were incubated at 35 ± 2 °C for 24 h to allow biofilm formation. After incubation, the culture medium was removed and replaced by sterile TSB. Then, VAN, HYDROGEL, and VAN-HYDROGEL were added through serial dilution to obtain concentration ranges of 32 to 0.5 µg/mL, 0.5 to 0.03%, and 32 µg/mL/0.05% to 1 µg/mL/0.03%, respectively, and the plates were incubated at 35 ± 2 °C for 24 h. After incubation, the crystal violet method described previously was performed. MBEC was determined as the lowest concentration capable of eradicating pre-formed biofilm. Independent experiments were performed in triplicate on different days.

##### Scanning Electron Microscopy of Biofilm

Initially, a MRSA ATCC 33591 suspension was adjusted to 0.5 on the McFarland scale and then exposed to VAN, HYDROGEL, and VAN-HYDROGEL at MBIC. Bacteria not exposed to treatment were used as a control for biofilm formation. Samples were prepared and placed on coverslips in the wells of a 24-well flat-bottom plate and incubated for 24 h at 35 ± 2 °C. After incubation, samples were washed three times with PBS buffer and fixed with a solution containing 2.5% glutaraldehyde and 4% paraformaldehyde in 0.1 M cacodylate buffer at pH 7.2 overnight. Then, 1% osmium tetroxide (OsO_4_) was added, with subsequent dehydration being performed through an increasing series of ethanol, proceeding to the critical point of drying with liquid CO_2_, followed by gold metallization and observation on a Jeol JSM-5600 scanning electron microscope at 15 kV [42]. Independent experiments were performed in triplicate on different days.

## 3. Results

### 3.1. Morphological Analysis of the HYDROGEL by SEM

**Figure 1:**
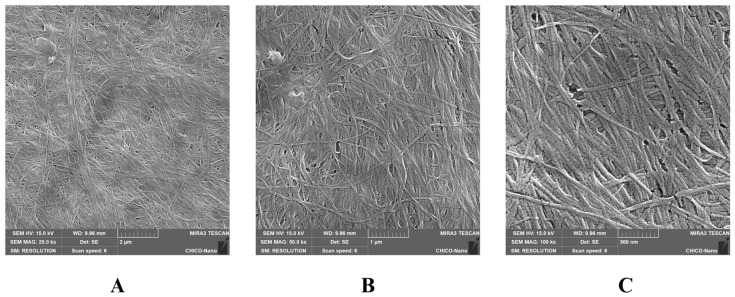
Bacterial cellulose HYDROGEL produced by *Zoogloea* sp. morphology by SEM at 25,000× (**A**), 50,000× (**B**), and 100,000× (**C**).

The HYDROGEL produced by *Zoogloea* sp. had a microporous structure, characterized by the three-dimensional conformation of cellulose fibers (Figure 1), allowing comprehensive adhesion of compounds.

### 3.2. Antibacterial Activity

VAN and VAN-HYDROGEL presented MICs of 0.5 to 1 μg/mL and ≤0.5 μg/mL/0.001% to 1 μg/mL/0.002% against *S. aureus* strains, respectively (Table 1). VAN and VAN-HYDROGEL also showed bactericidal activity with MBCs ranging from 2 to 16 μg/mL and ≤0.5 μg/mL/0.001% to 1 μg/mL/0.002% against *S. aureus* strains, respectively (Table 1).

Regarding *S. epidermidis* clinical isolates, the MIC values of VAN and VAN-HYDROGEL ranged from 1 to 2 μg/mL and 1 μg/mL/0.003% to 2 μg/mL/0.0078%, respectively (Table 2). VAN and VAN-HYDROGEL also showed bactericidal activity, with MBCs ranging from 4 to 8 μg/mL and 2 μg/mL/0.0078% to 4 μg/mL/0.0156% against *S. epidermidis* strains, respectively (Table 2). Thus, it was possible to observe that VAN-HYDROGEL exhibited bacteriostatic and bactericidal effects against *S. aureus* and *S. epidermidis* strains.

### 3.3. Time-Kill Assay

**Figure 2:**
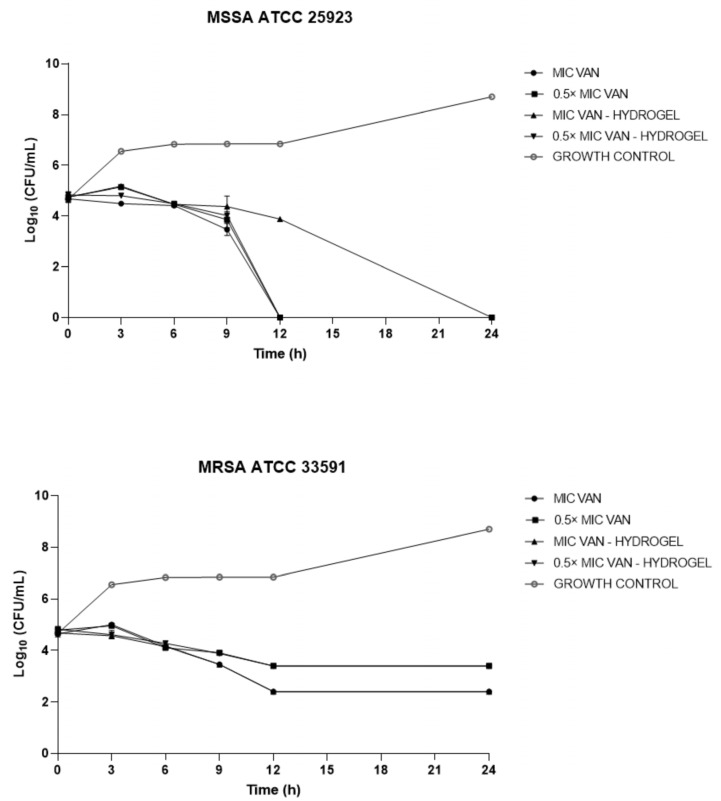
Time-kill curves of Vancomycin (VAN) and bacterial cellulose hydrogel produced by *Zoogloea* sp. containing vancomycin (VAN-HYDROGEL) at minimum inhibitory concentration (MIC) and 0.5 × MIC against methicillin-sensitive *Staphylococcus aureus* (MRSA ATCC 33591) and methicillin-sensitive *Staphylococcus aureus* (MSSA ATCC 25923).

The curves for the MSSA ATCC 25923 and MRSA ATCC 33591 strains showed different time-kill patterns (Figure 2). Both strains showed growth inhibition compared to the control group. For MSSA ATCC 25923, an exponential decrease in bacterial growth was observed after 9 h of exposure to VAN, which reduced by more than three logs compared to the initial culture, culminating in a bactericidal effect after 12 h of exposure. The MRSA ATCC 33591 strain demonstrated resistance to VAN, showing an exponential reduction in bacterial growth up to 12 h after exposure to the antimicrobial. Beyond 12 h, the number of bacteria cells remained linear, with no increase or decrease in colony-forming units (CFU). Through the incorporation of VAN into the HYDROGEL, it was observed that the antimicrobial activity profile remained unchanged.

### 3.4. Biofilm Formation

Most bacteria tested in this study showed strong biofilm production. Among the *S. aureus* strains, C137 was the only one that showed moderate biofilm production (Table 3). Regarding *S. epidermidis* strains, four were strong biofilm producers (ATCC 00016, C233, C266, and C271), while the other strains were moderate biofilm producers (Table 4).

### 3.5. Antibiofilm Activity

#### 3.5.1. Congo Red Agar Method

**Figure 3:**
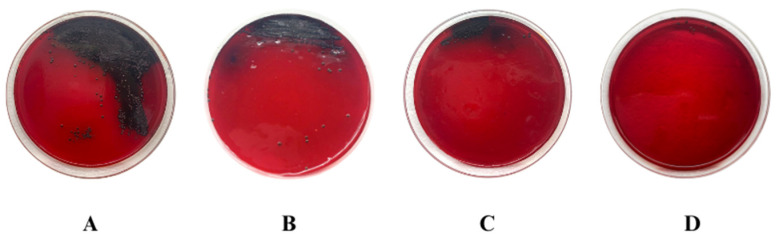
Effect of vancomycin (VAN), bacterial cellulose hydrogel produced by *Zoogloea* sp. (HYDROGEL), and bacterial cellulose hydrogel produced by *Zoogloea* sp. containing vancomycin (VAN-HYDROGEL) on the production of slime and exopolysaccharide (EPS) matrix of methicillin-resistant *Staphylococcus aureus* (MRSA ATCC 33591) by the Congo red agar method. (**A**) Untreated MRSA ATCC 33591 biofilm; (**B**) MRSA ATCC 33591 biofilm after treatment with VAN; (**C**) MRSA ATCC 33591 biofilm after treatment with HYDROGEL; (**D**) MRSA ATCC 33591 biofilm after treatment with VAN-HYDROGEL.

MRSA ATCC 33591 presented colonies with a blackish color and dry texture, as well as the production of slime and EPS matrix, characteristics presented by bacteria that produce biofilm, while performing the Congo red agar method (Figure 3A). After the incorporation of VAN (Figure 3B), HYDROGEL (Figure 3C), and VAN-HYDROGEL (Figure 3D) into the Congo red agar, a significant reduction in the production of slime and EPS matrix was observed, especially when the bacteria were exposed to VAN-HYDROGEL.

#### 3.5.2. Determination of Biofilm Inhibition and Eradication

Regarding the determination of antibiofilm activity using the crystal violet method, HYDROGEL and VAN showed an inhibitory effect on biofilm formation against most strains, but did not show biofilm eradication activity against methicillin-resistant *S. aureus* and *S. epidermidis* strains. However, we observed that biofilm eradication was enhanced after VAN incorporation into HYDROGEL (Table 5 and Table 6).

VAN presented MBICs ranging from 0.015 to >0.5 µg/mL, HYDROGEL from 0.25 to 0.5 µg/mL, and VAN-HYDROGEL from 0.015 µg/mL/0.01% to 0.5 µg/mL/0.5% against *S. aureus* strains (Table 5). For *S. epidermidis* isolates, VAN presented MBICs of 0.062 to 0.12 µg/mL, HYDROGEL of 0.03%, and VAN-HYDROGEL of 0.031 µg/mL/0.015% to 0.12 µg/mL/0.03% (Table 6).

Furthermore, VAN and HYDROGEL did not present a MBEC (>16 µg/mL and >0.5%, respectively), but VAN-HYDROGEL presented a MBEC of 016 µg/mL/0.5% for *S. aureus* strains (Table 5). Regarding *S. epidermidis* strains, VAN and HYDROGEL also did not present a MBEC (>16 µg/mL and >0.5%, respectively), but VAN-HYDROGEL showed MBECs of 8 µg/mL/0.25% to 16 µg/mL/0.5% (Table 6).

#### 3.5.3. Scanning Electron Microscopy of Biofilm

**Figure 4:**
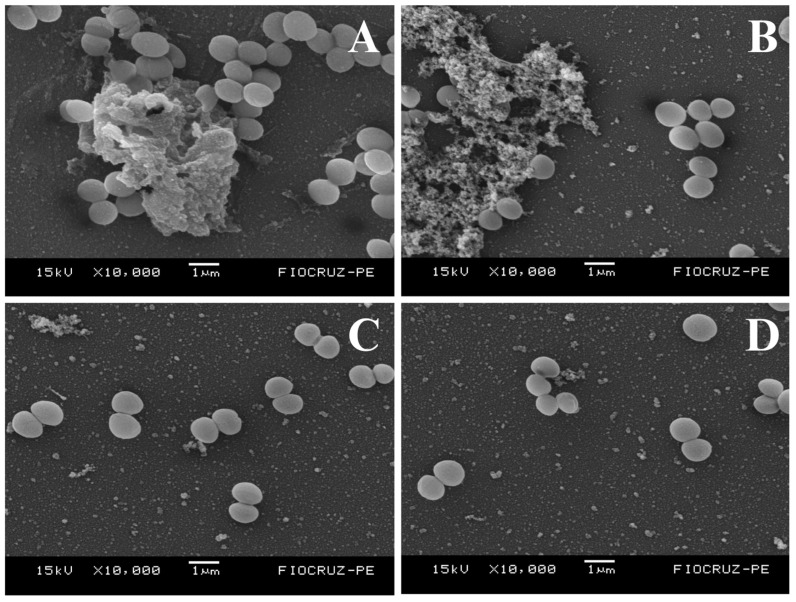
SEM of Methicillin-resistant *Staphylococcus aureus* (MRSA ATCC 33591) biofilm. (**A**) Untreated MRSA ATCC 33591 biofilm; (**B**) MRSA ATCC 33591 biofilm after treatment with vancomycin (VAN); (**C**) MRSA ATCC 33591 biofilm after treatment with bacterial cellulose hydrogel produced by *Zoogloea* sp. (HYDROGEL); (**D**) MRSA ATCC 33591 biofilm after treatment with bacterial cellulose hydrogel produced by *Zoogloea* sp. containing vancomycin (VAN-HYDROGEL).

Through SEM analysis, it was possible to observe the ability of VAN, HYDROGEL, and VAN-HYDROGEL to inhibit the formation of the *S. aureus* biofilm, although at different intensities (Figure 4). In Figure 4A, corresponding to the MRSA ATCC 33591 biofilm without any treatment, we observed an extensive area of EPS and many bacterial cells. When exposed to VAN at MBIC, the bacteria still expressed a high concentration of the EPS matrix (Figure 4B), while treatment with HYDROGEL demonstrated a significant reduction in the matrix (Figure 4C). Furthermore, VAN-HYDROGEL (Figure 4D) exhibited a more pronounced inhibition compared to VAN and HYDROGEL, suggesting an enhancement in the antibiofilm activity after the incorporation of VAN into HYDROGEL, revealing a greater capacity to inhibit EPS matrix expression and prevent biofilm formation.

## 4. Discussion

The hydrogel synthesized by *Zoogloea* sp. exhibits a structural resemblance to hydrogels previously reported in the literature, as emphasized by Gutierrez et al. [19]. The microporous architecture, illustrated by the three-dimensional arrangement of cellulose fibers, is due to the presence of hydroxyl groups on its surface, which favor intermolecular interactions and enable the adsorption of antimicrobial agents [20,21].

HYDROGEL by itself did not show any antibacterial activity, as described in the literature [43,44]. However, after incorporating VAN into HYDROGEL, there was a substantial increase in VAN activity, even demonstrating eight times greater activity. As already described in the literature, the antibacterial activity of VAN consists of interrupting the polymerization of peptidoglycans in the bacterial cell wall, as the drug binds to the terminal residue of the pentapeptide D-Ala-D-Ala, inhibiting the formation of cross-links between the pentapeptide and pentaglycine, thereby preventing the binding of new monomers. Thus, it is suggested that this mechanism of action is responsible for the antibacterial activity observed when VAN is incorporated into the HYDROGEL. This finding can be explained by the hydrogel’s ability to deliver the drug in a localized and targeted manner, promoting a prolonged release system, providing greater efficacy to the drug given the increase in its bioavailability to bacterial strains [45,46].

Gupta et al. [44] explored the antibacterial action of silver ions incorporated into BC hydrogel (BC-AgZ and BC-AgNO3), showing that the hydrogel did not exhibit activity, but BC-AgZ and BC-AgNO3 exhibited antimicrobial activity against strains of *P. aeruginosa* and *S. aureus*. The study highlighted the strong bactericidal activity of the formulations, describing the moist and responsive nature of bacterial cellulose hydrogels as an ideal feature for great biomaterial dressing destined for the treatment of chronically infected wounds. 

Additionally, Jiji et al. [45] also demonstrated the lack of antibacterial activity of BC hydrogel produced by the bacterium *Acetobacter xylinum*; nevertheless, after enriching it with thymol, it was possible to observe significant inhibition halos against strains of *S. aureus*, *Escherichia coli*, *P. aeruginosa*, and *Klebsiella pneumoniae*. Furthermore, Ao et al. [47] produced BC hydrogel using *A. xylinum* X-2 and enriched it with chitosan hydroxypropyltrimethyl ammonium chloride (HACC/BC). This study once again showed that BC hydrogel did not show antibacterial activity against several microorganisms, including *S. aureus* and MRSA. However, when these bacteria were exposed to HACC incorporated in BC, there was antibacterial activity.

Wichai et al. [7] incorporated sodium alginate, chitosan (Cs), and copper sulfate into BC membranes, forming the compound BC/AG/CS-Cu, which exhibited antibacterial action against MSSA ATCC 25923, MRSA ATCC 43300, *S. epidermidis* INCQS 00016 (ATCC 12228), *P. aeruginosa* ATCC 27853, *E. coli* ATCC 25922, *Acinetobacter baumannii* ATCC 19606, and vancomycin-resistant *Enterococci* (VRE) ATCC 51299. 

Wahid et al. [21] described the antibacterial activity of BC hydrogel formed by *Gluconacetobacter xylinus* enriched with CS. These formulations showed activity against *S. aureus* and *E. coli*, presenting an almost 90% reduction in the bacteria growth. Chanabodeechalermrung et al. [48] once again highlighted the benefits of the incorporation of molecules with antibacterial effects into hydrogels. In that study, the BC hydrogel developed with alginate and pectin incorporated with polyhexamethylene biguanide (PHMB) showed activity against strains of *S. aureus* ATCC 25923 and *P. aeruginosa* ATCC 27853. Therefore, all these studies have shown that isolated BC hydrogel does not have an antibiotic effect, but it is an important matrix for incorporating molecules with antibacterial activity.

Furthermore, the time-kill assay allowed a detailed evaluation and understanding of the action of free VAN and VAN-HYDROGEL. Evaluating antibacterial activity using this method is crucial to increase the chances of more effective therapy due to knowledge of the temporal effectiveness of the antimicrobial agent at different concentrations. Time-kill assays serve as a basis for in vivo studies, as they allow an in-depth understanding of the process of emergence of bacterial resistance within the population, providing crucial data for the development of more effective antimicrobial strategies [49,50].

The results showed that VAN was less effective against MRSA compared to MSSA. Over 9 h of exposure to VAN at different concentrations, MSSA was completely killed. Our results corroborate with the findings of a study carried out by Purwoningsih et al. [51] that performed a time-kill assay with VAN, linezolid, and ceftaroline against clinical isolates of MSSA and MRSA ATCC, and demonstrated greater efficacy of VAN against MSSA.

Thus, the present study provides experimental data of significant relevance with clinical implications that revolve around the assessment of HYDROGEL as a promising carrier for VAN. HYDROGEL stood out for not interfering with the specific antibacterial activity of VAN; therefore, the incorporation of VAN into cellulose gel emerges as a promising prospect, offering a series of benefits for topical applications [7], among them an increase in the viscosity of the formulation, providing a consistency that facilitates application to the skin. This attribute is essential to ensure that VAN remains in the desired application area, thus optimizing treatment efficacy.

Additionally, cellulose has a high capacity to promote adhesion of the gel to the epithelial surface, which will ensure a uniform distribution of VAN on the skin surface, favoring the homogeneous distribution of the antimicrobial in the treatment area [52,53,54]. Given these important physicochemical characteristics, BC and its combinations have been successfully applied in reconstructive medicine, attributable to their ability to promote wound healing [28,55]. Moreover, it is important to highlight that the hydrogel developed by *Zoogloea* sp. has undergone comprehensive evaluation in toxicity tests, as described by Pinto et al. [34], and safety studies conducted by Silva et al. [24]. Clinical research led by Silva et al. [26] confirmed not only the safety of the product but also its high capacity to promote wound healing in chronic venous ulcers in the lower extremities, showing significant reductions in wound area, improved healing rates, and positive patient outcomes when compared to standard dressings.

Regarding wound infections, it is known that bacteria that produce biofilm have a resistance profile to stress and antimicrobial exposure, since the microenvironment within the biofilm protects these bacteria and provides a safe place for resistance gene transfer between bacteria strains [56]. After the incorporation of VAN, HYDROGEL, and VAN-HYDROGEL into the Congo red agar, a significant reduction in slime and EPS matrix production was observed, especially when bacteria were exposed to VAN-HYDROGEL.

In a similar study, Ansari et al. [57] examined biofilm formation by *E. coli* and *Klebsiella* spp. on Congo red agar supplemented or not with silver nanoparticles (AgNPs). Control dishes showed black and dry colonies, indicating EPS production. However, in Congo red agar containing AgNPs, there was a decrease in bacterial growth and inhibition of EPS production. Furthermore, Ramachandran and Sangeetha [58] investigated the antibiofilm activity of AgNPs incorporated in Congo red agar against bacterial inocula of *E. coli*, *K. pneumoniae*, *P. aeruginosa*, *Proteus mirabilis*, and *A. baumannii*. Bacteria exposed to AgNPs exhibited less EPS production.

Santos et al. [39], when analyzing the production of the EPS matrix by *E. coli*, *K. pneumoniae*, *P. aeruginosa*, and *S. aureus* strains through the Congo red method, noticed that after the incorporation of AgNPs into Congo red agar, there was no evident EPS matrix formation, as only red colonies with a mucous texture appeared on the Petri dishes. Therefore, these studies indicate that agents with bactericidal properties, when incorporated into Congo red agar, can reduce both bacterial load and the production of EPS matrix during biofilm formation, as was evidenced in our study, especially after VAN incorporation into HYDROGEL.

Regarding the evaluation of antibiofilm activity using the crystal violet method, HYDROGEL and VAN showed an inhibitory effect on biofilm formation against most strains, but did not show biofilm eradication activity against methicillin-resistant *S. aureus* and *S. epidermidis* strains. However, we observed that biofilm eradication was enhanced after VAN incorporation into HYDROGEL (Table 5 and Table 6). Thus, from the results obtained, it is possible to observe that both HYDROGEL and VAN-HYDROGEL exhibit the effect of inhibiting biofilm formation, and VAN-HYDROGEL of eradicating formed biofilm.

Currently, the treatment of wounds infected caused by bacteria faces a major challenge, as most pathogens that affect the tissue are antibiotic-resistant and are biofilm producers. Therefore, a release system that acts both in inhibiting and eradicating the biofilm, such as VAN-HYDROGEL, is necessary, as these characteristics will promote efficiency in the treatment of wounds that no longer respond to therapy with conventional antibiotics, enabling regeneration of tissue and reducing resistance. Thus, HYDROGEL is becoming an excellent vehicle for antibiotics to combat MDR bacteria [59,60,61].

Other studies have also reported the enhancement in the antibiofilm effect of molecules when incorporated into BC hydrogel. He et al. [62] incorporated tonic acid (TA) and magnesium chloride (MgCl_2_) into BC and reported significant inhibition of biofilm production from *P. aeruginosa* and *S. aureus* strains (above 60% inhibition), with BC-TA having the best inhibitory effect against these strains.

Zhang et al. [27], when evaluating the antibiofilm activity of tannic acid incorporated into BC hydrogel against *S. aureus* and *P. aeruginosa*, also observed an enhancement in the activity of this acid after incorporation into BC hydrogel through a significant reduction in the number of bacteria observed in electron microscopy (above 75% inhibition).

Fasiku et al. [63] treated MRSA biofilms by applying hydrogen peroxide and antimicrobial peptides to a Cs-based hydrogel. In this study, the authors observed that the groups treated with hydrogen peroxide and antimicrobial peptides showed eradication of MRSA biofilms in a range that varied from 13% to 75%. In a similar way, Zmejkoski et al. [64] evaluated the antibiofilm activity of BC hydrogels enriched with Cs and Cs nanoparticles. This evaluation was carried out with Gram-positive bacteria, such as *Streptococcus agalactiae*, β-hemolytic *Streptococcus*, *S. aureus*, and *Enterococcus faecalis*, and with Gram-negative bacteria, such as *E. coli*, *K. pneumoniae*, *P. mirabilis*, and *P. aeruginosa*. All compounds showed antibiofilm activity when incorporated in BC hydrogel against all tested bacteria, reducing biofilm formation by up to 90%.

Pandian et al. [65], while testing AgNPs incorporated into N,O-carboxymethyl Cs-based self-healing hydrogel against *E. coli*, *S. aureus*, and *P. aeruginosa* strains, observed biofilm eradication from 68.86 to 83.22%. However, it is important to highlight that the study did not record any activity of the isolated hydrogel, in contrast with the results obtained in our study, in which HYDROGEL not only demonstrated biofilm eradication activity when incorporated with VAN, but also exhibited inhibitory activity when tested alone.

Therefore, all of these studies have shown that both BC hydrogel and other biopolymer-based hydrogels, such as CS, have emerged as promising vehicles in the treatment of biofilms. It is also important to highlight that, to our knowledge, no studies have shown elimination capacity of the bacterial biofilm compared to what we observed in HYDROGEL.

The EPS matrix plays a crucial role in *S. aureus* and *S. epidermidis* biofilm formation, as it maintains structural integrity, facilitating adhesion, communication between cells, the transport of molecules, and nutrition within the biofilm. As a result, available treatments are partially ineffective against strains of *Staphylococcus* spp., due to the difficulty in penetrating the biofilm caused by the expression of EPS [66,67]. In this context, the development of systems that facilitate drug entry into the biofilm and inhibit its expression, as is the case of VAN-HYDROGEL, is necessary.

The SEM analysis showed varying degrees of effectiveness in inhibiting *S. aureus* biofilm formation by VAN, HYDROGEL, and VAN-HYDROGEL. While all treatments reduced biofilm formation, VAN-HYDROGEL exhibited the most pronounced inhibition, indicating enhanced antibiofilm activity compared to VAN and HYDROGEL alone.

In a similar study, Zhang et al. (2020) [27] observed changes in the morphology of *S. aureus* and *P. aeruginosa* through microscopy after treatment with BC hydrogel enriched with TA and Mg, called BC-TA-Mg. This treatment was able to irreversibly damage the cellular structure of the bacteria, in addition to inhibiting bacterial aggregation and EPS production. However, the isolated hydrogel did not show biofilm inhibitory capacity, only eradication when incorporated with Ta and Mg, which differentiates it from HYDROGEL.

Furthermore, studies involving other biopolymers, such as Cs, were reported by Muthuchamy et al. [68]. When evaluating the treatment of biofilms with Cs compounds enriched with graphene through SEM analysis, it was possible to notice characteristics such as irregular surface and membrane collapse, among other evidence that highlighted the biopolymer antibiofilm effect. Thus, these studies have demonstrated how hydrogels and biopolymer compounds, such as BC, can enhance antimicrobial treatment through the incorporation of antibacterial agents.

## 5. Conclusions

HYDROGEL preserved the antibacterial potential of VAN, in addition to enhancing its antibiofilm activity, preventing biofilm from forming, and eradicating formed biofilm of MDR *S. aureus* and *S. epidermidis* strains. It is worth mentioning that HYDROGEL by itself also showed effectiveness in preventing biofilm formation against these strains.

Therefore, this nanobiotechnological formulation presents itself as a promising dressing material for future in vivo studies regarding its topical use in the treatment of wounds infected by MDR bacteria.

## Figures and Tables

**Table 1:** Antibacterial activity of HYDROGEL, VAN, and VAN-HYDROGEL against *S. aureus* strains.

Bacteria	HYDROGEL		VAN		VAN-HYDROGEL	
	MIC	MBC	MIC	MBC	MIC	MBC
	(%)		μg/mL		VAN (μg/mL)/HYDROGEL (%)	
MRSA ATCC 33591	>0.5	>0.5	1	8	0.5/0.002	1/0.002
C047	>0.5	>0.5	0.5	2	≤0.5/0.001	1/0.002
C074	>0.5	>0.5	0.5	4	≤0.5/0.001	1/0.002
C115	>0.5	>0.5	0.5	4	≤0.5/0.001	2/0.004
C128	>0.5	>0.5	0.5	16	≤0.5/0.001	2/0.004
C137	>0.5	>0.5	0.5	8	≤0.5/0.001	2/0.004

MIC: Minimum inhibitory concentration; MBC: Minimum bactericidal concentration; MSSA: Methicillin-sensitive *Staphylococcus aureus*; MRSA: Methicillin-resistant *Staphylococcus aureus*; ATCC: American Type Culture Collection; C047, C074, C115, C128, and C137: Methicillin-resistant *Staphylococcus aureus* clinical isolates; HYDROGEL: Bacterial cellulose hydrogel produced by *Zoogloea* sp.; VAN: Vancomycin; VAN-HYDROGEL: Bacterial cellulose hydrogel produced by *Zoogloea* sp. containing vancomycin.

**Table 2:** Antibacterial activity of HYDROGEL, VAN, and VAN-HYDROGEL against *S. epidermidis* strains.

Bacteria	HYDROGEL		VAN		VAN-HYDROGEL	
	MIC	MBC	MIC	MBC	MIC	MBC
	(%)		μg/mL		VAN (μg/mL)/HYDROGEL (%)	
*S. epidermidis* ATCC 12228 (INCQS 00016)	>0.5	>0.5	1	4	1/0.003	2/0.0078
C233	>0.5	>0.5	2	4	2/0.0078	4/0.0156
C266	>0.5	>0.5	2	4	2/0.0078	4/0.0156
C271	>0.5	>0.5	2	4	2/0.0078	4/0.0156
C276	>0.5	>0.5	2	4	2/0.0078	4/0.0156
C277	>0.5	>0.5	2	4	2/0.0078	4/0.0156
C281	>0.5	>0.5	2	8	2/0.0078	4/0.0156
C387	>0.5	>0.5	2	4	2/0.0078	4/0.0156
C389	>0.5	>0.5	1	4	2/0.0078	4/0.0156
C417	>0.5	>0.5	2	4	2/0.0078	2/0.0078

MIC: Minimum inhibitory concentration; MBC: Minimum bactericidal concentration; ATCC: American Type Culture Collection; INCQS: National Institute of Health Quality Control of the Oswaldo Cruz Foundation (Fiocruz); C233, C266, C271, C276, C277, C281, C387, C389, and C417: Methicillin-resistant clinical isolates of *Staphylococcus epidermidis*; HYDROGEL: Bacterial cellulose hydrogel produced by *Zoogloea* sp.; VAN: Vancomycin; VAN-HYDROGEL: Bacterial cellulose hydrogel produced by *Zoogloea* sp. containing vancomycin.

**Table 3:** Classification of biofilm production of *S. aureus* strains.

Bacteria	Biofilm Production
MSSA ATCC 25923	Strong
MRSA ATCC 33591	Strong
C047	Strong
C074	Strong
C115	Strong
C128	Strong
C137	Moderate

MSSA: Methicillin-sensitive *Staphylococcus aureus*; MRSA: Methicillin-resistant *Staphylococcus aureus*; ATCC: American Type Culture Collection; C047, C074, C115, C128, and C137: Methicillin-resistant clinical isolates of *Staphylococcus aureus*.

**Table 4:** Classification of biofilm production of *S. epidermidis* strains.

Bacteria	Biofilm Production
*S. epidermidis* ATCC 12228 (INCQS 00016)	Strong
C233	Strong
C266	Strong
C271	Strong
C276	Moderate
C277	Moderate
C281	Moderate
C387	Moderate
C389	Moderate
C417	Moderate

ATCC: American Type Culture Collection; INCQS: National Institute of Health Quality Control of the Oswaldo Cruz Foundation (Fiocruz); C233, C266, C271, C276, C277, C281, C387, C389, and C417: Methicillin-resistant clinical isolates of *Staphylococcus epidermidis*.

**Table 5:** Antibiofilm activity of HYDROGEL, VAN, and VAN-HYDROGEL against *S. aureus.*

	HYDROGEL		VAN		VAN-HYDROGEL	
Bacteria	MBIC	MBEC	MBIC	MBEC	MBIC	MBEC
	(%)		μg/mL		VAN (μg/mL)/HYDROGEL (%)	
MSSA ATCC 25923	0.5	>0.5	0.015	>16	0.015/0.01	16/0.5
MRSA ATCC 33591	0.5	>0.5	>0.5	>16	0.12/0.12	16/0.5
C047	0.25	>0.5	>0.5	>16	0.25/0.25	16/0.5
C074	0.5	>0.5	>0.5	>16	0.5/0.5	16/0.5
C115	0.25	>0.5	>0.5	>16	0.25/0.25	16/0.5
C128	0.25	>0.5	>0.5	>16	0.12/0.12	16/0.5
C137	0.5	>0.5	>0.5	>16	0.25/0.25	16/0.5

MBIC: Minimum biofilm inhibitory concentration; MBEC: Minimum biofilm eradication concentration; MSSA: Methicillin-sensitive *Staphylococcus aureus*; MRSA: Methicillin-resistant *Staphylococcus aureus*; ATCC: American Type Culture Collection; C047, C074, C115, C128, and C137: Methicillin-resistant clinical isolates of *Staphylococcus aureus*; HYDROGEL: Bacterial cellulose hydrogel produced by *Zoogloea* sp.; VAN: Vancomycin; VAN-HYDROGEL: Bacterial cellulose hydrogel produced by *Zoogloea* sp. containing vancomycin.

**Table 6:** Antibiofilm activity of HYDROGEL, VAN, and VAN-HYDROGEL against *S. epidermidis*.

	HYDROGEL		VAN		VAN-HYDROGEL	
Bacteria	MBIC	MBEC	MBIC	MBEC	MBIC	MBEC
	(%)		μg/mL		VAN (μg/mL)/HYDROGEL (%)	
*Staphylococcus epidermidis* ATCC 12228 (INCQS 00016)	0.03	>0.5	0.062	>16	0.031/0.015	8/0.25
C233	0.03	>0.5	0.12	>16	0.12/0.03	16/0.5
C266	0.03	>0.5	0.12	>16	0.12/0.03	16/0.5
C271	0.03	>0.5	0.12	>16	0.12/0.03	16/0.5
C276	0.03	>0.5	0.12	>16	0.12/0.03	8/0.25
C277	0.03	>0.5	0.12	>16	0.12/0.03	16/0.5
C281	0.03	>0.5	0.12	>16	0.12/0.03	16/0.5
C387	0.03	>0.5	0.12	>16	0.12/0.03	16/0.5
C389	0.03	>0.5	0.12	>16	0.06/0.03	16/0.5
C417	0.03	>0.5	0.12	>16	0.12/0.03	8/0.25

MBIC: Minimum biofilm inhibitory concentration; MBEC: Minimum biofilm eradication concentration; ATCC: American Type Culture Collection; INCQS: National Institute of Health Quality Control of the Oswaldo Cruz Foundation (Fiocruz); C233, C266, C271, C276, C277, C281, C387, C389, and C417: Methicillin-resistant clinical isolates of *Staphylococcus epidermidis*; HYDROGEL: Bacterial cellulose hydrogel produced by *Zoogloea* sp.; VAN: Vancomycin; VAN-HYDROGEL: Bacterial cellulose hydrogel produced by *Zoogloea* sp. containing vancomycin.

## Data Availability

Data are contained within the article.
